# Addition of Bevacizumab to Chemotherapy and Its Impact on Clinical Efficacy in Cervical Cancer: A Systematic Review and Meta-Analysis

**DOI:** 10.3390/pharmacy12060180

**Published:** 2024-12-01

**Authors:** Aleena Shahzad, Anees ur Rehman, Tehnia Naz, Muhammad Fawad Rasool, Alisha Saeed, Saba Rasheed, Sadia Shakeel, Saleh Karamah Al-Tamimi, Rabia Hussain

**Affiliations:** 1 Department of Pharmacy Practice, Faculty of Pharmacy, Bahauddin Zakariya University, Multan 60800, Pakistan; alenashahzad@gmail.com (A.S.); tehnianaz83@gmail.com (T.N.); fawadrasool@bzu.edu.pk (M.F.R.); alishasaeed21@gmail.com (A.S.);; 2 Dow College of Pharmacy, Dow University of Health Sciences, Karachi 74200, Pakistan; sadia.shakeel@duhs.edu.pk; 3 Faculty of Pharmacy, University of Aden, Aden 11712, Yemen; 4 Discipline of Social and Administrative Pharmacy, School of Pharmaceutical Sciences, Universiti Sains Malaysia, George Town 11800, Penang, Malaysia

**Keywords:** bevacizumab, cervical cancer, efficacy, metastasis, monoclonal antibody, meta-analysis

## Abstract

*Background and Objectives*: Cervical cancer is the third leading cause of cancer-related mortality in females. One of the most successful therapeutic modalities to date is suppressing vascular endothelial growth factor (VEGF)-mediated angiogenesis. Bevacizumab is a monoclonal antibody that targets VEGF-A. The outcomes for cervical cancer patients treated with bevacizumab in combination with platinum-based chemotherapy have been explored in several studies. This study aimed to assess the impact of bevacizumab on progression-free survival (PFS) and overall survival (OS) in patients with metastatic cervical cancer. *Materials and Methods*: This systematic review was registered in PROSPERO (CRD42023456755). Following PRISMA guidelines, a comprehensive literature search on PubMed and Google Scholar identified 28 studies meeting the inclusion criteria. The outcomes of interest were PFS and OS. The statistical analysis computed hazard ratios (HRs) with 95% confidence intervals (CIs). The study also included a subgroup analysis by cervical cancer stage. *Results*: The pooled analysis revealed that bevacizumab-based therapy significantly improved both PFS with HR 0.77 (95% CI: 0.58–0.96; *p* < 0.01; I^2^ = 58%) and OS with HR 0.63 (95% CI: 0.45–0.89; *p* < 0.01; I^2^ = 41%) in cervical cancer patients. Subgroup analysis by stage of cervical cancer demonstrated better efficacy of bevacizumab in metastatic stage IVB cervical cancer patients indicated by HR for PFS (0.69, 95% CI: 0.54–0.79; *p* < 0.01) and HR for OS (0.57, 95% CI: 0.46–0.73; *p* < 0.01). *Conclusions*: Bevacizumab exhibits a significant increase in PFS and OS, underscoring the efficacy of anti-angiogenesis therapy in cervical cancer, particularly in stage IVB metastatic cervical cancer patients.

## 1. Introduction

Cervical cancer is the fourth most frequent cancer in females globally, and according to reports, it was the third leading cause of cancer-related mortality in females in 2020, accounting for 342,000 deaths [1,2]. The patterns of incidence and mortality vary widely worldwide, with over 85% of the burden falling on low- and middle-income nations [3]. Southeast Asia, Sub-Saharan Africa, and Latin America have the highest diagnosis and mortality rates [4,5].

Human papillomaviruses (HPVs) of various subtypes are the main trigger of cervical cancer, accounting for more than 90% of cases [6]. Of these cases, 71% are caused by HPV types 16 and 18, while the remaining 19% are caused by HPV types 31, 33, 45, 52, and 58 [7]. The division of HPV subtypes varies somewhat between squamous and adenocarcinoma of the cervix [6]. Since HPV has a significant role in the development of cervical cancer, preventive measures such as cytologic screening and HPV vaccinations have been developed and the incidence rates have largely been controlled in developed countries. But still, people in developing countries present with advanced disease [8].

Clinical staging guidelines provided by the International Federation of Gynecology and Obstetrics (FIGO) are the basis for cervical cancer treatment [8]. Most of the early cervical cancers are treated by surgical procedures such as radical trachelectomy, simple and radical hysterectomy, and lymphadenectomy with or without cervical conization [9]. For patients diagnosed with stage IB2 and above, concurrent chemoradiation (a combination of high-dose-rate intracavitary brachytherapy and platinum-based chemoradiation) is considered the gold standard of therapy [5,8]. The FIGO classification of stage IVA or IVB denotes metastatic cervical cancer, signifying the spread of the disease from the cervix to the rectum, bladder, or lymph nodes [10]. Approximately 15% of patients experience distant metastases [8].

The patients who develop metastatic disease (FIGO stage IVB) or nonresectable localized recurrence are left with limited treatment options, and only 5–15% of them are expected to survive for five years [11]. Tumor metastasis requires vasculature, which is supported by angiogenesis, a mechanism that accelerates cervical cancer progression caused by HPV [5,12]. For patients with metastatic or recurrent cervical cancer, single-agent cisplatin has proven to be an effective treatment [6]. The majority of patients, however, experience a median life expectancy of seven to twelve months due to short responses to chemotherapy doublets containing platinum, which lead to a rapid reduction in quality of life and early death [5].

One of the most successful therapeutic modalities to date is suppressing angiogenesis, which has recently improved the treatment of cervical cancer [13]. Vascular endothelial growth factor (VEGF) is a vital tumor angiogenesis mediator, adversely correlated with survival and directly with disease severity [14]. Since the primary cause of cervical cancer progression is VEGF-driven angiogenesis, anti-angiogenic therapy has recently emerged as a promising strategy in the treatment of persistent, metastatic, or recurrent cervical cancer [15]. Patients with cervical cancer who have elevated VEGF levels are treated with bevacizumab, a monoclonal antibody that targets VEGF-A, which prevents signal transmission through VEGF receptors 1 and 2 and tends to suppress its activity [8,16]. Its objective is to regulate tumor vasculature, reduce interstitial fluid pressure (IFP), and restrict tumor oxygenation [8]. The first phase III trial, GOG 240, examined the efficacy of chemotherapy (topotecan/paclitaxel or cisplatin/paclitaxel) with or without bevacizumab, and this study revealed a significant improvement in overall survival (OS) with this targeted treatment [13]. While the GOG 240 study demonstrated significant enhancements in overall survival and progression-free survival, there was no substantial decline in health-related quality of life [17]. As a result, the United States Food and Drug Administration approved bevacizumab in August 2014 for the treatment of patients with recurrent, persistent, or metastatic cervical cancer [18]. The outcomes and safety considerations for patients with cervical cancer treated with bevacizumab in combination with platinum-based chemotherapy have been explored in several other studies [11].

The significance of this current meta-analysis is underscored by the lack of prior systematic reviews and meta-analyses examining the impact of bevacizumab on progression-free survival (PFS) and overall survival (OS) in cervical cancer across a range of chemotherapy regimens. Thus, the purpose of this study was to analyze the existing evidence on the efficacy of bevacizumab combined with chemotherapy and its impact on PFS and OS in patients with metastatic cervical cancer. This work fills a major gap in the literature by conducting a pioneering investigation of bevacizumab’s collective efficacy in the context of multiple chemotherapy regimens.

## 2. Materials and Methods

### 2.1. Protocol Registration

This systematic review was registered in PROSPERO (International Prospective Register of Systematic Reviews) under the registration number CRD42023456755.

### 2.2. Search Strategy

Following the guidelines for preferred reported items in systematic reviews and meta-analyses (PRISMA) [19], we conducted a meta-analysis of clinical trials and cohort studies and sought relevant research in PubMed and Google Scholar databases. The search strategy was meticulously designed using specific keywords and Boolean operators to ensure a comprehensive screening of relevant studies. For PubMed, the following search terms were used: (“bevacizumab”[MeSH Terms] OR “bevacizumab”[All Fields]) AND (“uterine cervical neoplasms”[MeSH Terms] OR “cervical cancer”[All Fields]) AND (“efficacy”[MeSH Terms] OR “treatment outcome”[MESH Terms]). The screening process involved three stages: the initial screening of titles, followed by the evaluation of abstracts, and the final assessment of full-text articles for eligibility. The references of the included studies were also searched. The search method was restricted to English language. Exclusively considered were the studies focusing on the administration of bevacizumab in the treatment of cervical cancer in adult females aged 18 years and older.

### 2.3. Inclusion and Exclusion Criteria

The inclusion and exclusion criteria were followed in the screening of the studies. The decisions about which studies to include and exclude were made in consultation with the co-author. The inclusion criteria comprised (1) original research (clinical trials and cohort studies) featuring patients with cervical cancer, (2) patients receiving bevacizumab-based chemotherapy, (3) studies providing comprehensive patient data, and (4) reporting clearly defined outcomes or endpoints of PFS and OS. The exclusion criteria encompassed studies that reported outcomes beyond survival metrics (such as adverse events, treatment response, economic factors, and quality of life measures), expert opinions, editorials, abstracts, literature reviews, case reports, and research that did not evaluate the impact of bevacizumab.

### 2.4. Outcomes

The outcomes of our interest in this meta-analysis were progression-free survival (PFS) and overall survival (OS). PFS refers to the time from the initiation of treatment until the first evidence of disease progression or metastasis. In other words, it is the period in which the patient lives with the disease without it getting worse. On the other hand, OS describes the period from the moment of diagnosis (or the commencement of therapy) till the moment of death. It is used to assess how well a treatment is working.

### 2.5. Study Selection and Data Extraction

The process of finding and choosing relevant studies was completed separately by two authors. Discussion and agreement were used to settle any differences or conflicts in their evaluations. To limit the possibility of bias in the literature included in this meta-analysis, a third author was consulted to see whether a consensus could be achieved, assuring an impartial selection of studies. The retrieved data from the included studies were assembled by two independent authors using a standard template sheet created specifically for this study. Data extraction included the following: the first author’s name and the year of publication, the study design (study setting, duration, participants, details of randomization, and therapy given), recruitment variables (inclusion and exclusion criteria), follow-up, study outcomes, statistical analysis, and results (patient demographics, median follow-up, outcomes expressed as hazard ratios [HRs], and *p*-values).

### 2.6. Risk of Biasness

The Newcastle–Ottawa Scale (NOS), as suggested by the Cochrane Collaboration, was established to assess the probability of bias in the included research. This scale is an eight-item checklist that evaluates quantitative studies and is divided into three sections: (1) selection, (2) comparability, and (3) ascertainment of exposure/outcome. Selection allows for a maximum of four points, comparability allows for two points, and exposure/outcome allows for three points [20]. Studies are graded on a scale of 0 to 9, with a score of 0–3 suggesting low quality and a high risk of bias, a score of 4–6 indicating moderate quality and a moderate risk of bias, and a score of 7–9 indicating good or high quality and a low risk of bias [21].

### 2.7. Statistical Analysis

Statistical analyses were executed using Stata version 12.0 (Stata Corp, College Station, TX, USA), employing the fixed-effect model to compute pooled hazard ratios (HRs) along with 95% confidence intervals (CIs). The I^2^ statistic test was used to examine statistical heterogeneity, with I^2^ values of 25%, 50%, and 75% indicating low, moderate, and high heterogeneity, respectively. Subgroup analyses based on the stage of cervical cancer were performed to evaluate the impact of bevacizumab on PFS and OS in stage IVB cervical cancer. Forest plots were generated for each efficacy measure to consolidate the findings of this meta-analysis into a singular numerical outcome. For all statistical analyses, a *p*-value of less than 0.01 was considered statistically significant. We used a full dataset for our statistical analysis, and none of the studies included had any missing data.

## 3. Results

**Figure 1:**
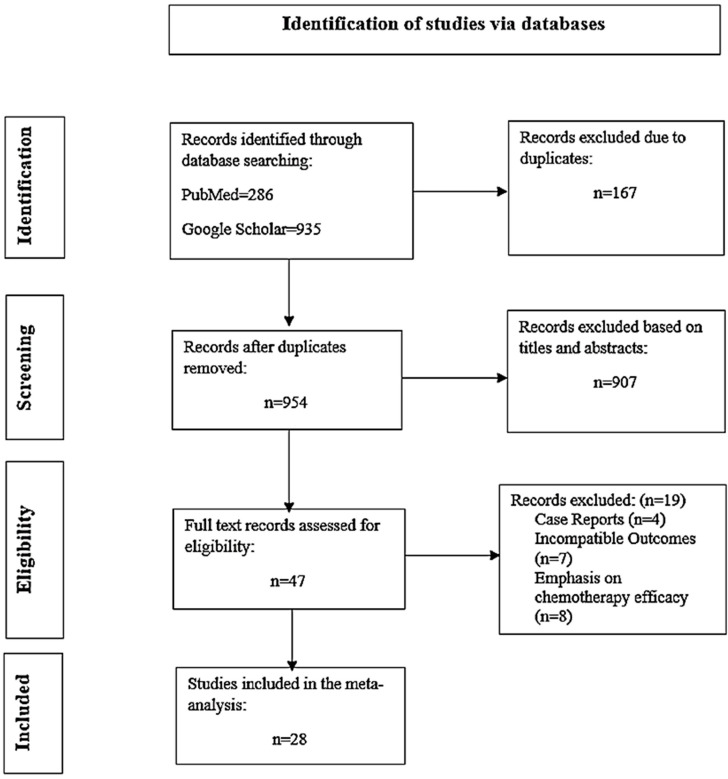
PRISMA flow diagram of the study selection process.

The search yielded 935 results on Google Scholar and 286 results on PubMed. There were 167 duplicate studies. Following a screening of the titles and abstracts, 47 studies were selected for full-text analysis, of which 28 met the predetermined inclusion criteria and were included in the analysis. The remaining studies were excluded for the following reasons: four were case reports [22,23,24,25], seven focused on outcomes other than survival [10,26,27,28,29,30,31], and eight primarily concentrated on the efficacy of chemotherapy rather than bevacizumab [15,32,33,34,35,36,37,38]. The detailed flow diagram of the study selection process is presented in Figure 1.

### 3.1. Characteristics of the Included Studies

The characteristics of the included studies are presented in Table 1. Among the 28 included studies, there were 8 clinical trials, including 6 phase II non-randomized trials and 2 phase III randomized controlled trials (RCTs); 19 retrospective studies; and 1 prospective study. The dataset comprised a total of 3087 cases, with 1274 assigned to the control group and 1813 allocated to the experimental group. The sample sizes ranged from a minimum of 6 to a maximum of 452.

### 3.2. Demographics

Sex was not taken into account in demographics as cervical cancer affects females only. Patients were diagnosed histologically in most of the studies. Patients receiving bevacizumab varied in age from 33 to 67.2 years. A total of 1318 patients receiving bevacizumab had a diagnosis of squamous cell carcinoma with metastatic or stage IVB cervical cancer in 497 patients and recurrence being predominant in 876 patients. The most frequent kind of local recurrence was pelvic, and radiation or concomitant platinum-based chemoradiotherapy was the usual therapy for pelvic recurrence. Among the patients, 993 had a performance score of 0, while 731 had a performance score of 1. The following combinations of chemotherapy were common in the studies for the experimental group: cisplatin and paclitaxel with bevacizumab were administered to 869 patients in 15 studies, carboplatin and paclitaxel with bevacizumab were administered to 593 patients in 11 studies, and topotecan and paclitaxel with bevacizumab were administered to 237 patients in 3 studies. The standard chemotherapy in the control group was administered in a median of 3–10 cycles, while bevacizumab in the experimental group was administered in a median of 2–12 cycles. The dose of bevacizumab was 15 mg/kg in most studies, while only a few administered a lower dose of 7.5 mg/kg. Certain studies incorporated a follow-up element, with the standard follow-up visit occurring every three months for two years and subsequently every six months for three years or until disease progression. PET-CT or MRI was performed at every follow-up visit to assess the tumor. Table 2 depicts the characteristics of patients included in the studies.

### 3.3. Efficacy Measures:

#### 3.3.1. Efficacy of Bevacizumab on PFS in Cervical Cancer

**Figure 2:**
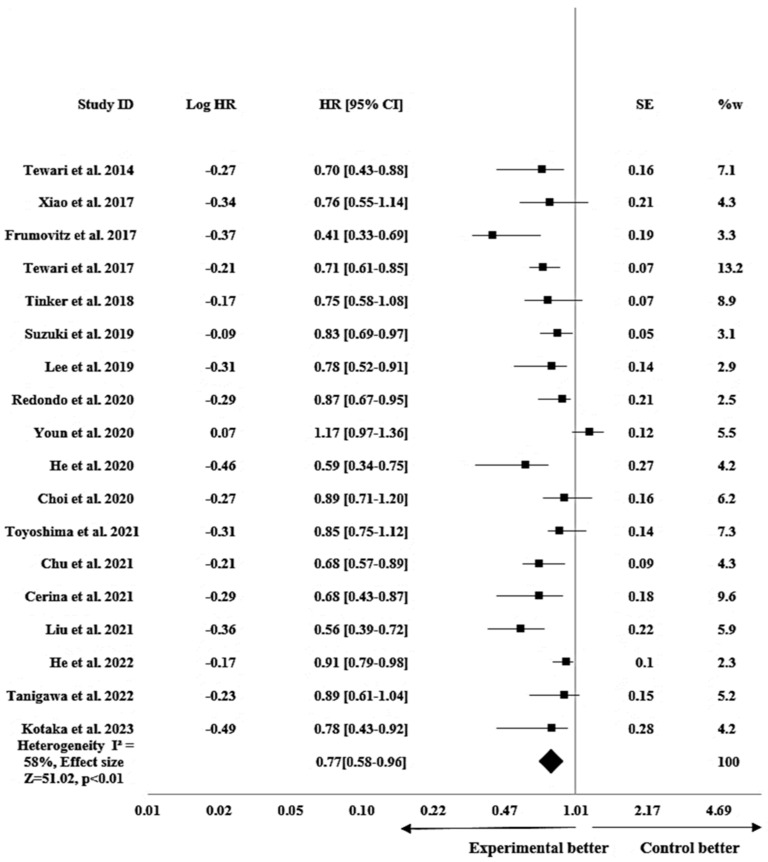
Forest plot showing efficacy of bevacizumab on PFS in cervical cancer patients. The meta-analysis results are illustrated as pooled hazard ratios (HRs) with 95% confidence intervals (CIs) for PFS across the included studies. Each square represents an individual study’s effect size [1,2,11,12,17,18,45,47,49,51,52,53,55,56]. The horizontal lines show 95% CIs, and the diamond represents the overall pooled estimate (0.77 [0.58–0.96]). The I^2^ statistic (58%) indicates heterogeneity across the studies.

The pooled analysis of PFS revealed that bevacizumab-based chemotherapy significantly improved PFS in patients with cervical cancer with HR 0.77 (95% CI: 0.58–0.96; *p* < 0.01). The results of the fixed effects model showed a heterogeneity I^2^ of 58% and an effect size Z of 51.02 as shown in Figure 2. PFS was assessed in 24 studies with a median PFS of 4.3 to 16 months for patients receiving bevacizumab-based chemotherapy (experimental group) and a median PFS of 4 to 13.5 months for those receiving chemotherapy only (control group). The median follow-up in the studies ranged from 9 to 38 months for patients in the experimental group and 10.9 to 43.3 months for patients in the control group.

#### 3.3.2. Efficacy of Bevacizumab on PFS in Metastatic (Stage IVB) Cervical Cancer Patients

**Figure 3:**
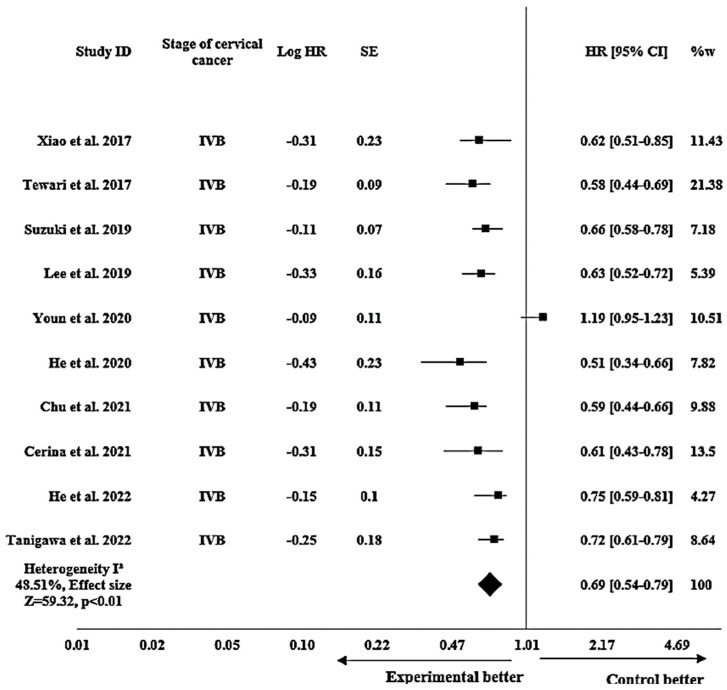
Forest plot showing efficacy of bevacizumab on PFS in metastatic (stage IVB) cervical cancer patients. The meta-analysis results are illustrated as pooled hazard ratios (HRs) with 95% confidence intervals (CIs) for PFS in stage IVB patients across the included studies. Each square represents an individual study’s effect size [2,5,11,18,43,47,49,53,55,56]. The horizontal lines show 95% CIs, and the diamond represents the overall pooled estimate (0.69 [0.54–0.79]). The I^2^ statistic (48.51%) indicates heterogeneity across the studies.

In the subgroup analysis focusing solely on metastatic (stage IVB) cervical cancer patients, the pooled HR for PFS was calculated as 0.69 (95% CI: 0.54–0.79; *p* < 0.01), and the outcomes revealed a heterogeneity I^2^ of 48.51% and an effect size Z of 59.32 as shown in Figure 3.

#### 3.3.3. Efficacy of Bevacizumab on OS in Cervical Cancer

**Figure 4:**
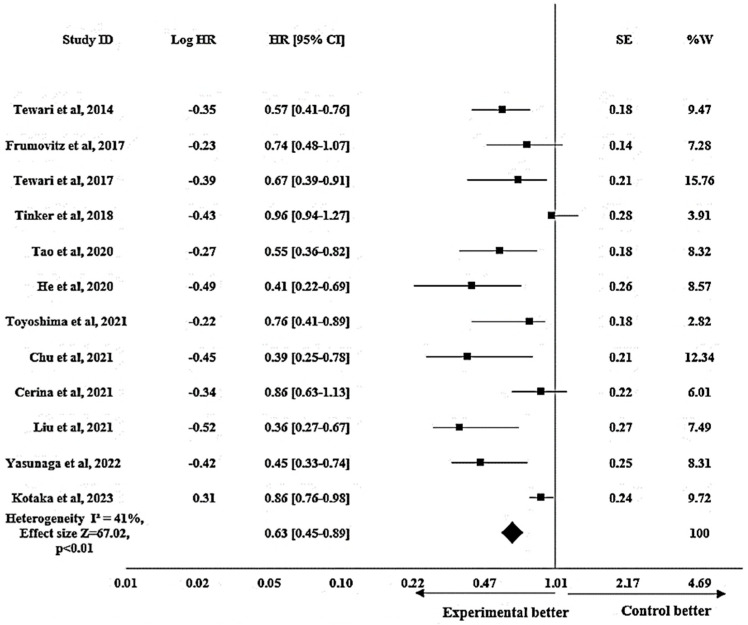
Forest plot showing efficacy of bevacizumab on OS in cervical cancer patients. The meta-analysis results are illustrated as pooled hazard ratios (HRs) with 95% confidence intervals (CIs) for OS patients across the included studies. Each square represents an individual study’s effect size [1,2,5,12,14,44,45,48,49,52,53,57]. The horizontal lines show 95% CIs, and the diamond represents the overall pooled estimate (0.63 [0.45–0.89]). The I^2^ statistic (41%) indicates heterogeneity across the studies.

The pooled analysis of OS exhibited an improvement in OS related to the use of bevacizumab-based chemotherapy with HR 0.63 (95% CI: 0.45–0.89; *p* < 0.01). A heterogeneity I^2^ of 41% and an effect size Z of 67.02 were displayed by the fixed effects model. The results of the pooled analysis on OS are depicted in Figure 4. OS was assessed in 26 studies with a median OS of 5.1 months to 34 months for patients receiving bevacizumab-based chemotherapy (experimental group) and a median OS of 7 to 29 months for patients receiving chemotherapy only (control group).

#### 3.3.4. Efficacy of Bevacizumab on OS in Metastatic (Stage IVB) Cervical Cancer Patients

**Figure 5:**
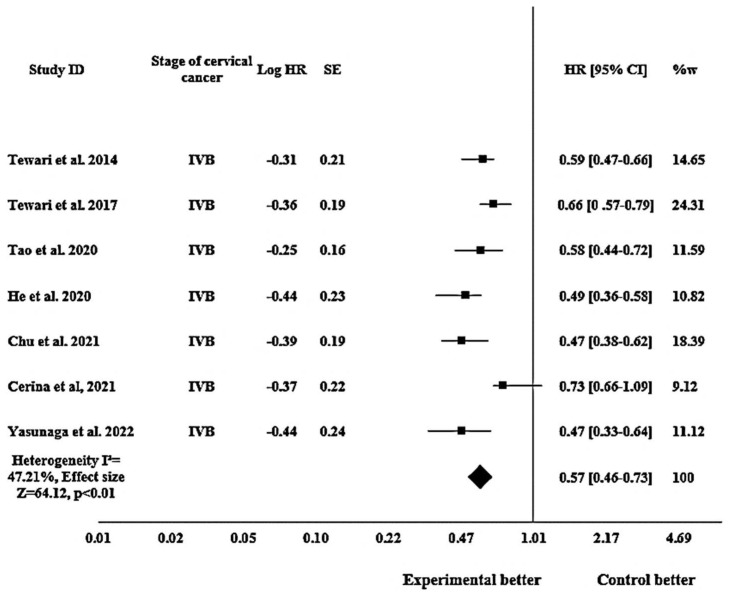
Forest plot showing efficacy of bevacizumab on OS in metastatic (stage IVB) cervical cancer patients. The meta-analysis results are illustrated as pooled hazard ratios (HRs) with 95% confidence intervals (CIs) for OS in stage IVB patients across the included studies. Each square represents an individual study’s effect size [2,5,14,48,49,53,57]. The horizontal lines show 95% CIs, and the diamond represents the overall pooled estimate (0.57 [0.46–0.73]). The I^2^ statistic (47.21%) indicates heterogeneity across the studies.

In the subgroup analysis focusing solely on metastatic (stage IVB) cervical cancer patients, the pooled HR for OS was reported as 0.57 (95% CI: 0.46–0.73; *p* < 0.01) exhibiting a heterogeneity I^2^ of 47.21% and an effect size Z of 64.12, as shown in Figure 5.

### 3.4. Risk of Bias Assessment

The included studies showed a score range of 6 to 9 on the Newcastle–Ottawa Scale (NOS). Significantly, 19 of the included studies showed excellent quality, with scores in the top range of the spectrum (7–9) exhibiting a low risk of bias. The remaining nine studies displayed a moderate risk of bias, with a quality score of 6. The aggregate mean Newcastle–Ottawa Scale (NOS) score for the included studies was 7, indicating a low risk of bias and demonstrating good quality throughout the studies included in the analysis. The risk of bias among included studies is given in Table 3.

## 4. Discussion

This systematic review and meta-analysis provide a substantial contribution to the field of cervical cancer treatment. A thorough synthesis of the data from various trial designs presents a comprehensive picture of the efficacy of bevacizumab. This study incorporated several research types, which improved the robustness and generalizability of our results, in contrast to previous studies. Moreover, the novelty of this study is highlighted by the lack of prior reviews or meta-analyses assessing the impact of bevacizumab on PFS and OS in cervical cancer patients incorporating such a broad range of trial designs and chemotherapeutic regimens. Our results suggest directions for future study and guide clinical practice and treatment guidelines.

The substantial therapeutic advantages of combining bevacizumab with chemotherapy to mitigate angiogenesis in patients with cervical cancer, particularly in terms of PFS and OS, are affirmed by the results of this systematic review and meta-analysis. Based on the value of significance reported in these investigations, 18 of the 28 included studies—2 phase III RCTs, 3 phase II trials, 12 retrospective studies, and 1 prospective study—were analyzed for PFS. Similarly, 12 studies—2 phase III RCTs, 9 retrospective studies, and 1 prospective study—were assessed for OS. A subgroup analysis focused on stage IVB of cervical cancer in which 10 studies for PFS and 7 studies for OS were thoroughly analyzed.

To quantify bevacizumab’s treatment impact on survival in the included studies, the results were translated into HRs along with 95% CIs. Bevacizumab-based chemotherapy was superior to chemotherapy alone when the HR was less than 1, which showed a link between bevacizumab and lengthening survival rates. For PFS, the pooled HR indicated that as compared with the control group, the therapy group had a 23% lower risk of disease or tumor progression, showing a survival enhancement. The analysis revealed a moderate heterogeneity among the studies, and the observed effect size suggested a significant influence of bevacizumab on PFS. Additionally, the pooled PFS effect was statistically significant (*p* < 0.01). All things considered, these numbers lend credence to bevacizumab’s possible efficacy in raising PFS in cases of cervical cancer.

Parallel to the observations in PFS, the pooled data revealed a noteworthy enhancement in OS within the bevacizumab arm, showcasing a 37% reduction in the risk of death in the therapy group compared with the control group. There appeared to be some variation throughout the studies, as indicated by the heterogeneity, and a substantial effect size demonstrated a considerable impact of bevacizumab on OS. The observed effect was statistically significant (*p* < 0.01). As a whole, this finding illustrated how well bevacizumab worked to raise OS in cervical cancer. Even though we noted an improvement in OS, we were reluctant to draw precise inferences about the treatment of cervical cancer in terms of OS due to the lack of statistical significance in the included studies. These conclusions need to be supported by extra research.

In comparing the studies analyzed for PFS, Lee et al. [11] and Kotaka et al. [1] demonstrated the closest results to the pooled effect, whereas Frumovitz et al. [44] demonstrated the most detached figure. This discrepancy might probably be attributed to the diverse patient group; our study included a wider spectrum of cervical cancer patients, while this study concentrated on individuals with small cell carcinoma of the cervix. Similarly, among the studies analyzed for OS, Tewari et al. [5] showed the closest result to the pooled effect, while Liu et al. [12] had the most disparate outcome. The provided dose of bevacizumab was most likely the reason for this discrepancy. The experimental group in this study received a dose of 7.5 mg/kg, whereas the dose used in other included studies was 15 mg/kg.

The subgroup analysis revealed promising results of bevacizumab in terms of PFS and OS in patients with stage IVB cervical cancer, thus depicting enhanced efficacy of bevacizumab particularly in stage IVB cervical cancer. For PFS, the pooled HR indicated a 31% reduction in the risk of disease progression for patients with stage IVB cervical cancer, exposed to bevacizumab. The results exhibited a moderate heterogeneity, and the observed effect size indicated a substantial impact of bevacizumab on PFS in stage IVB cervical cancer. Suzuki et al. 2019 [47] showed the closest result to the pooled effect, while He et al. 2020 [49] showed a detached figure. Similarly, the analysis revealed a favorable impact of bevacizumab on OS in stage IVB cervical cancer patients. The calculated HR demonstrated a 43% reduction in the risk of mortality among these patients. The observed heterogeneity indicated a moderate variation among the included studies, but the outstanding effect size signified a considerable impact of bevacizumab on the OS outcome in this population of patients. Tao et al. 2020 [48] aligned most closely with the pooled effect while Chu et al. 2021 [53] and Yasunaga et al. 2022 [57] showed a slight deviation. The persistent statistical significance of our findings suggested that the observed benefit of bevacizumab on PFS and OS was stable in patients with stage IVB cervical cancer.

By assessing each study’s quality using the NOS, the level of confidence in every single study included in this meta-analysis was established. Nineteen of the included studies had a quality score of 7 to 9, indicating high quality and low bias. The remaining nine studies scored 6, considered satisfactory, but still provided useful information regarding bevacizumab’s efficacy in cervical cancer. The variation in these quality ratings provided the analysis with more breadth and sturdiness.

As far as we are aware, this is the largest meta-analysis of this kind that has been performed to date since rather than comparing bevacizumab with particular chemotherapeutic agents, we used a more comprehensive strategy by comparing it with various chemotherapy regimens, where the combination of cisplatin and paclitaxel was the most prevalent in majority studies. Our study’s strengths stem from the fact that we took into account retrospective, prospective, non-randomized, and randomized controlled trials. Using a variety of trial designs, we were able to compile an extensive body of evidence regarding the impact of bevacizumab on cervical cancer. Additionally, by computing HRs for both PFS and OS, we thoroughly evaluated bevacizumab’s efficacy in cervical cancer. Finally, concentrating on bevacizumab specifically gave our study more significance and specificity, setting it apart from other studies in the field. As a result, our study has considerable significance since it adds to the body of knowledge regarding the efficacy of bevacizumab in the treatment of cervical cancer. A recently published study demonstrated that the addition of bevacizumab to standard chemotherapy significantly improved both PFS and OS in patients with colorectal cancer [58]. This finding highlights the broader applicability of bevacizumab across various tumor types by targeting angiogenesis effectively.

While this study offered valuable insights, there were certain limitations. Variables like the differences in patient demographics and examined treatment methods might have impacted the generalizability of our findings. Furthermore, many studies, including phase II trials and certain retrospective studies, did not include a control arm, which indicated that the treatment outcomes of bevacizumab and a control group were not directly compared in these studies. Almost all of the analyzed studies offered convincing proof of bevacizumab’s efficacy, but for PFS, just one retrospective study by Youn et al. [18] did not yield significant findings. Notwithstanding the aforementioned constraints, our study offered valuable insights into the efficacy of bevacizumab in cervical cancer.

The clinical implications of our meta-analysis are noteworthy. Our results can be used by clinicians to customize treatment regimens for cervical cancer patients, possibly using bevacizumab in individualized strategies. Optimizing treatment techniques can be achieved by identifying predictive variables for bevacizumab response, and treatment regimens can be refined by evaluating the drug’s effectiveness in different combinations and dose schedules. The strong evidence produced could have an impact on clinical recommendations, standardizing the use of bevacizumab in protocols. All things considered, our meta-analysis offers practical recommendations for clinical practice, patient care, and continuing research in cervical cancer treatment.

## 5. Conclusions

Our extensive pooled analysis highlights the notable therapeutic advantages associated with bevacizumab-based chemotherapy in the context of cervical cancer treatment. The results demonstrate significant improvements in both PFS and OS. Subgroup analysis reaffirms these findings, indicating the enhanced efficacy of bevacizumab especially in stage IVB metastatic cervical cancer patients. Our study contributes valuable insights, emphasizing the efficacy of bevacizumab in enhancing survival outcomes for patients with cervical cancer. In conclusion, our findings underscore the ability of bevacizumab as a potential component in the evolving landscape of cervical cancer treatment strategies. Bevacizumab is a promising innovation in the treatment of cervical cancer, and the results of our study highlight its importance in enhancing survival, especially in patients with stage IVB cervical cancer. Inspired by our positive findings, this strategy is essential for developing personalized treatment strategies, thus enhancing the prognosis and quality of life in cervical cancer patients.

## Figures and Tables

**Table 1:** Summaries of the included studies.

Author	Study Design	Stage of Cervical Cancer	No. of Patients		Therapy Protocol		Outcomes		PFS (Median, Months)			OS (Median, Months)		
			C	E	C	E	Primary	Secondary	C	E	*p*-Value	C	E	*p*-Value
Wright et al., 2006 [39]	Retrospective	IB2, IIB, IIIB	-	6	-	5-Fluorouracil + Bev and oral capecitabine + Bev	OS, PFS	-	-	4.3	-	-	5.1	-
Monk et al., 2009 [40]	Phase II trial	I–IV	-	46	-	One or two cytotoxic regimens + Bev	PFS	OS	-	3.40	-	-	7.29	-
Zighelboim et al., 2013 [41]	Phase II trial	I–IV	-	27	-	Cisplatin + topotecan + Bev	PFS	OS	-	7.1	-	-	13.2	-
Tewari et al., 2014 [14]	Phase III RCT	I–IVB	225	227	Cisplatin + paclitaxel and topotecan + paclitaxel	Cisplatin + paclitaxel + Bev and + topotecan + paclitaxel + Bev	OS	PFS	5.9	8.2	-	13.3	17.0	0.004
Schefter et al., 2014 [42]	Phase II trial	IB–IIIB	-	49	-	Cisplatin + Bev + pelvic RT and BT	-	OS	-	-	-	-	81.3%	-
Xiao et al., 2017 [43]	Retrospective	IVB	15	15	Cisplatin + paclitaxel	Cisplatin + paclitaxel + Bev and docetaxel + nedaplatin + Bev	PFS	-	7	10	0.023	-	-	-
Frumovitz et al., 2017 [44]	Retrospective	IB1–IV	21	13	Available chemotherapy regimens	Topotecan + paclitaxel + Bev	OS, PFS	-	4	7.8	0.001	9.4	9.7	0.13
Godoy-Ortiz et al., 2017 [13]	Retrospective	I–IVB	-	27	-	Cisplatin/carboplatin + paclitaxel + Bev	OS, PFS	-	-	9, 6	-	-	21, 5	-
Tewari et al., 2017 [5]	Phase III RCT	IVB	225	227	Cisplatin + paclitaxel and topotecan + paclitaxel	Cisplatin + paclitaxel + Bev and topotecan + paclitaxel + Bev	OS	PFS	6	8.2	0.0002	13.3	16.8	0.007
Tinker et al., 2018 [45]	Retrospective	IB1/2–IVB	-	27	-	Carboplatin + paclitaxel + Bev	OS, PFS	-	-	5.3	-	-	11	-
Fagotti et al., 2018 [46]	Retrospective	IVB	-	15	-	Cisplatin/carboplatin + paclitaxel + Bev	OS	-	-	-	-	-	13	-
Suzuki et al., 2019 [47]	Phase II trial	IVB	-	34	-	Carboplatin + paclitaxel + Bev	-	OS, PFS	-	9	-	-	26	-
Lee et al., 2019 [11]	Retrospective	I–IVB	-	57	-	Cisplatin + paclitaxel + Bev	OS, PFS	-	-	9.8	-	-	15.3	-
Tao et al., 2020 [48]	Retrospective	IVB	161	127	Carboplatin + paclitaxel	Carboplatin + paclitaxel + Bev	OS	-	-	-	-	2–29	2–31	0.038
Redondo et al., 2020 [17]	Single-arm phase II trial	I–IVB	-	150	-	Carboplatin + paclitaxel + Bev	-	OS, PFS	-	10.9	-	-	25	-
Youn et al., 2020 [18]	Retrospective	IVB	30	11	Carboplatin + paclitaxel or cisplatin + topotecan + RT	Cisplatin + paclitaxel + Bev + RT	OS, PFS	-	46.7%	45.5%	0.22	72.9%	81.8%	0.57
He et al., 2020 [49]	Retrospective	IVB	134	130	Cisplatin + paclitaxel	Cisplatin + paclitaxel + Bev	OS, PFS	-	8.58	11.34	0.000	11.73	17.74	0.002
Ercelep et al., 2020 [50]	Retrospective	IB–IVB	-	64	-	Cisplatin/carboplatin + paclitaxel + Bev	OS, PFS	-	-	8	-	-	23	-
Choi et al., 2020 [51]	Retrospective	I–IVB	92	71	Cisplatin + paclitaxel + ifosfamide followed by ifosfamide + mesna	Cisplatin + paclitaxel + Bev	OS, PFS	-	12	13.1	0.353	-	-	-
Toyoshima et al., 2021 [52]	Prospective	I–IV	4	15	Cisplatin/ carboplatin + paclitaxel + Bev without Bev maintenance	Cisplatin/carboplatin + paclitaxel + Bev + Bev single maintenance	OS, PFS	-	7	12	0.6805	21	Not Reached	0.0132
Chu et al., 2021 [53]	Retrospective	IVB	122	124	Cisplatin + paclitaxel	Cisplatin + paclitaxel + Bev	OS, PFS	-	7.9	9.2	<0.001	12.3	16.4	0.001
Yang et al., 2021 [54]	Retrospective	IIB to IIIC2, or IVB	-	64	-	Cisplatin (DDP) + Bev + RT and cisplatin + docetaxel + Bev	OS	-	-	-	-	-	87.2%	-
Cerina et al., 2021 [2]	Retrospective	IVB	62	67	Cisplatin + paclitaxel, cisplatin + 5-Fluorouracil, ifosfamide + cisplatin, topotecan + paclitaxel or cisplatin + gemcitabine	Cisplatin + paclitaxel + Bev	OS	PFS	5.4	10.6	0.027	15.5	27.0	0.389
Liu et al., 2021 [12]	Retrospective	I–IV	43	21	Cisplatin + paclitaxel	Cisplatin + paclitaxel + Bev	OS, PFS	-	17.7%	51.0%	0.003	23.2%	55.5%	0.005
He et al., 2022 [55]	Retrospective	IVB	-	65	-	Bev + pemetrexed	OS, PFS	-	-	6.6	-	-	10.6	-
Tanigawa et al., 2022 [56]	Single-arm phase II trial	IVB	-	69	-	Carboplatin + paclitaxel + Bev	PFS	OS	-	11.3	-	-	Not Reached	-
Yasunaga et al., 2022 [57]	Retrospective	I–IVB	59	31	Carboplatin + paclitaxel	Carboplatin + paclitaxel + Bev	OS	-	-	-	-	12	31	0.069
Kotaka et al., 2023 [1]	Retrospective	I–IVB	81	34	Cisplatin/ carboplatin/ nedaplatin + paclitaxel + Bev without Bev maintenance	Cisplatin/ carboplatin/ nedaplatin + paclitaxel + Bev with Bev maintenance	PFS, OS	-	9.0	16.0	0.041	29.0	34.4	0.374

Key. C—control group; Bev—bevacizumab; BT—brachytherapy; E—experimental group; OS—overall survival; PFS—progression-free survival; RCT—randomized controlled trial; RT—radiation therapy.

**Table 2:** Characteristics of patients in the included studies.

Author	Recruitment Variables		Patient Characteristics			
	Inclusion Criteria	Exclusion Criteria	Tumor Stage	Histopathology	Race	HPV Infection
Wright et al., 2006 [39]	Recurrent disease	-	IB2: 3 IIB: 2 IIIB: 1	SCC: 4 ADC: 1 PDC: 1	White: 3 Black: 3	-
Monk et al., 2009 [40]	GOG PS 0/1, normal body parameters, prior chemotherapy	Non-SCC tumors, bleeding, wounds, infection, CV or CNS disease	I–IV	ASC: 3 SCC: 43	Asian: 3 African American: 4 Hispanic: 6 American Indian: 1 White: 32	-
Zighelboim et al., 2013 [41]	Age ≥ 18 years, no prior chemotherapy, GOG score 0/1, normal body parameters	Infection, bleeding or wounds, CNS or CV disease, history of surgery, or any malignancy in 5 years	I: 9 II: 8 III: 8 IV: 2	SCC: 18 ADC: 9	White: 23 African American: 4	-
Tewari et al., 2014 [14]	GOG score 0/1, adequate renal, hepatic, and bone marrow function	Curative pelvic exenteration candidates, bleeding, and non-healing wounds	I–IVB	ADC: 86 ASC: 44 SCC: 310 Other: 12	Not Black: 392 Black: 60	-
Schefter et al., 2014 [42]	Pelvic node metastases and/or tumor size ≥ 5 cm, Zubrod PS of 0–2, normal body parameters	Surgery, bleeding, thromboembolic events	IB: 8 IIA: 1 IIB: 31 IIIA: 1 IIIB: 8	SCC: 39 ADC: 8 ASC: 2	Hispanic or Latino: 3 Not Hispanic or Latino: 41 Unknown: 5	-
Xiao et al., 2017 [43]	Karnofsky scores ≥ 70 points, normal ECGs	History of serious organ/system disease, bleeding or circulatory collapse	IVB	SCC: 25 ADC: 5	-	-
Frumovitz et al., 2017 [44]	Small cell cervix cancer, prior chemoradiotherapy for first recurrence	Large cell or carcinoid cervical tumors, first recurrence treated with radiation or surgery for oligometastatic disease	IB: 21 IIB: 2 IIIB: 5 IV: 4	-	White: 21 Black: 3 Hispanic: 4 Asian: 1 Unknown: 5	-
Godoy-Ortiz et al., 2017 [13]	ECOG scores 0/1	-	I–IVB	SCC: 21 ADC: 3 Others: 3	-	-
Tewari et al., 2017 [5]	GOG score 0/1, normal body functions, urine protein to creatinine ratio < 1	Candidates for curative therapy via pelvic exenteration, non-healing wounds or active bleeding, receiving chemotherapy for recurrence	IVB	SCC: 310 ADC: 86 Other: 56	White: 351 African American: 60 Asian: 19 Pacific Islander: 1 Others: 21	-
Tinker et al., 2018 [45]	Recurrent, persistent disease	ECOG scores > 3, uncontrolled hypertension, major surgery, pregnancy/ breastfeeding, bleeding, diathesis	Metastatic: 7 IIIA/B: 6 IIA/B: 10 IB1/2: 4	SCC: 24 ADC: 2 Unknown: 1	-	-
Fagotti et al., 2018 [46]	Age 70 years or less, normal functions, no prior non-basal cell carcinoma	Disease progression during treatment, previous or concurrent malignancies, any severe infection	IVB	SCC: 11 ADC: 2 Clear cells: 2	-	-
Suzuki et al., 2019 [47]	GOG score 0/1, normal body parameters and functions	Evident malignancies, wounds and bleeding, infections, prior therapy and surgery, pregnancy	IVB: 9	SCC: 21 ASC: 2 ADC: 7 Others: 4	-	-
Lee et al., 2019 [11]	Persistent, recurrent, or metastatic disease	Previous treatment with Bev	I–IVB	SCC: 37 ADC: 12 ASC: 1 Others: 2	-	-
Tao et al., 2020 [48]	Age > 18 years, not suitable for surgery/radiation, normal body parameters and functions	-	IVB: 77	SCC: 198 ADC: 68 ASC: 22	Han Chinese: 262 Mongolian: 21 Tibetan: 3 Uighur: 2	-
Redondo et al., 2020 [17]	Non-measurable disease, age ≥ 18 years, life expectancy ≥ 3 years, ECOG score 0/1	Ongoing bladder/rectal involvement, prior chemotherapy, history of fistula/GI perforation, known HIV infection	I: 19 II: 44 III: 47 IVA: 6 IVB: 34	-	Hispanic or Latino: 50 Not Hispanic or Latino: 91 Not reported/unknown: 9	-
Youn et al., 2020 [18]	Initial diagnosis of stage IVB cervical cancer, distant metastases	Dual primary cancers, non-radiotherapy group, no follow-up data	IVB	SCC: 38 ADC: 3	-	Negative: 10 Positive: 31
He et al., 2020 [49]	Postmenopausal females with advanced disease, age 55–75 years, history of HPV infections, GOG score 0/1, normal body functions	Severe organ failure, non-healing wounds, risk of bleeding, coma	IVB: 144	SCC: 150 ADC: 91 Other: 23	-	-
Ercelep et al., 2020 [50]	Persistent, recurrent, or metastatic disease	-	IB: 14 II: 13 III: 9 IVA: 11 IVB: 18	SCC: 57 ADC: 5 ASC: 2	-	-
Choi et al., 2020 [51]	Measurable disease progression, ECOG score 0–2, normal body functions	Prior pelvic exenteration, non-cervical malignancy within 5 years	CIS–II: 127 III–IV: 28 Unknown: 8	SCC: 109 Other: 54	-	-
Toyoshima et al., 2021 [52]	Age 20–75 years, ECOG score 0–2, normal functions	Prior anti-VEGF therapy, intestinal obstruction, non-healing wounds, history of cerebrovascular accident, risk of bleeding, pregnancy, other malignancies	I: 2 II: 5 III: 5 IV: 3	SCC: 9 ADC: 4 ASC: 1 SCC+ ADC: 1	-	-
Chu et al., 2021 [53]	Postmenopausal Chinese females with previously untreated advanced disease, GOG PS 0/1	Prior use of targeted drugs, chemotherapy/RT, organ failure, malignancies, active bleeding	IVB	SCC: 151 ADC: 71 ASC: 24	-	-
Yang et al., 2021 [54]	Pelvic relapse after surgery, Zubrod PS 0–2	Thromboembolic events/bleeding within the previous 6 months	IIB–IIIC: 48 IVB-Ln only: 10 6	SCC: 58 Non-SCC: 6	-	-
Cerina et al., 2021 [2]	Treatment with TCB as a first-line setting	-	IVB	SCC: 97 ADC: 23 Other: 9	-	-
Liu et al., 2021 [12]	Confirmed recurrence, normal body functions	-	I: 16 II: 13 III: 11 IV: 24	SCC: 38 ADC: 17 ASC: 9	-	-
He et al., 2022 [55]	Disease progression or relapse after first-line therapy, normal body functions, ECOG score 0–2, life expectancy ≥ 3 months	Allergy to pemetrexed or Bev, organ function impairment, malignancies	IVB	SCC: 49 ADC: 16	-	-
Tanigawa et al., 2022 [56]	Age ≥ 20 years, ECOG score ≤ 1, normal organ functions	-	IVB: 23	SCC: 46 ADC: 20 ASC: 3	-	-
Yasunaga et al., 2022 [57]	ECOG status 0–2, normal body parameters For Bev: controlled BP, proteinuria	For Bev: peritoneal dissemination with colonic invasion, deep venous thrombosis, and complication of active inflammatory bowel disease	I–IVB	SCC: 61 ADC: 23 Other: 6	-	-
Kotaka et al., 2023 [1]	No prior Bev combination therapy	Platinum-paclitaxel Chemotherapy + Bev for the third or subsequent relapse	I: 11 II: 15 III: 57 IV: 32	SCC: 77 ADC: 21 Other: 17	-	-

Key. ADC—adenocarcinoma; ASC—adenosquamous carcinoma; Bev—bevacizumab; BP—blood pressure; CV—cardiovascular; CNS—central nervous system; ECOG—Eastern cooperative oncology group; GOG—gynecologic oncology group; PDC—poorly differentiated carcinoma; PS—performance status; RT—radiation therapy; SCC—squamous cell carcinoma; TCB—cisplatin + paclitaxel + bevacizumab.

**Table 3:** Risk of bias among included studies.

Author	Study Design	Selection	Comparability	Outcome/ Exposure	NOS Score	Quality	Risk of Bias
Wright et al., 2006 [39]	Retrospective	***	**	*	6	Moderate	Moderate
Monk et al., 2009 [40]	Phase II trial	***	**	*	6	Moderate	Moderate
Zighelboim et al., 2013 [41]	Phase II trial	***	**	*	6	Moderate	Moderate
Tewari et al., 2014 [14]	Phase III RCT	****	**	**	8	High	Low
Schefter et al., 2014 [42]	Phase II trial	***	**	*	6	Moderate	Moderate
Xiao et al., 2017 [43]	Retrospective	****	**	*	7	High	Low
Frumovitz et al., 2017 [44]	Retrospective	****	*	***	8	High	Low
Godoy-Ortiz et al., 2017 [13]	Retrospective	***	**	***	8	High	Low
Tewari et al., 2017 [5]	Phase III RCT	****	**	**	8	High	Low
Tinker et al., 2018 [45]	Retrospective	***	**	***	8	High	Low
Fagotti et al., 2018 [46]	Retrospective	***	**	***	8	High	Low
Suzuki et al., 2019 [47]	Phase II trial	***	**	*	6	Moderate	Moderate
Lee et al., 2019 [11]	Retrospective	***	**	***	8	High	Low
Tao et al., 2020 [48]	Retrospective	****	**	*	7	High	Low
Redondo et al., 2020 [17]	Single arm phase II trial	***	**	*	6	Moderate	Moderate
Youn et al., 2020 [18]	Retrospective	****	**	**	8	High	Low
He et al., 2020 [49]	Retrospective	****	**	**	8	High	Low
Ercelep et al., 2020 [50]	Retrospective	***	*	**	6	Moderate	Moderate
Choi et al., 2020 [51]	Retrospective	****	**	**	8	High	Low
Toyoshima et al., 2021 [52]	Prospective	****	**	*	7	High	Low
Chu et al., 2021 [53]	Retrospective	****	**	***	9	High	Low
Yang et al., 2021 [54]	Retrospective	***	*	**	6	Moderate	Moderate
Cerina et al., 2021 [2]	Retrospective	****	**	**	8	High	Low
Liu et al., 2021 [12]	Retrospective	****	**	**	8	High	Low
He et al., 2022 [55]	Retrospective	***	**	**	7	High	Low
Tanigawa et al., 2022 [56]	Single arm phase II trial	***	**	*	6	Moderate	Moderate
Yasunaga et al., 2022 [57]	Retrospective	****	**	**	8	High	Low
Kotaka et al., 2023 [1]	Retrospective	***	*	***	7	High	Low

## Data Availability

Data are available on request from the corresponding author.
